# Las políticas sanitarias en Córdoba: entre ideas y concreciones, 1930-1955

**DOI:** 10.1590/S0104-59702024000100033

**Published:** 2024-07-15

**Authors:** Karina Inés Ramacciotti

**Affiliations:** i Universidad Nacional de Quilmes; Consejo Nacional de Investigaciones Científicas y Técnicas. Ciudad Autónoma de Buenos Aires – Argentina karinaramacciotti@gmail.com

*La salud como problema provincial*, de María José Ortiz Bergia (2022), es un libro que refleja un gran trabajo teórico e historiográfico. Nos permite adentrarnos en las torsiones del sistema federal de la Argentina y en cómo impactó dicha característica de la Constitución Nacional en el financiamiento, diseño y administración del subsistema de salud público cordobés a partir de los años 1930.

Sin lugar a dudas, este trabajo se suma a otros que vienen analizando las capacidades de los estados a la hora de implementar las políticas sanitarias durante la primera mitad del siglo XX en Argentina (Ramacciotti, 2009; Bacolla, 2016; Fernández, 2017; Jerez, 2016; Hirschegger, 2016). Ortiz Bergia (2022) los refiere y dialoga constantemente con ellos, posibilitando comprender el desigual mapa de la estructura sanitaria de la Argentina y, como ella misma sostiene, su investigación, si bien no es un análisis generalizable a otros casos, permite elaborar un esquema descriptivo y explicativo de los procesos históricos. En consecuencia, si bien María José Ortiz Bergia escoge como estudio de caso la provincia de Córdoba, su propuesta metodológica habilita en el futuro a estudiar otros casos.

*La salud como problema provincial* nos invita a encontrar, en el pasado, líneas de procesos sociosanitarios que, sin lugar a dudas, llegan hasta la actualidad. Allí podemos comprender las tensiones entre las acciones nacionales y provinciales a la hora de distribuir vacunas, organizar campañas sanitarias y expandir la estructura asistencial. También el libro se detiene en la manera en que los procesos de salud y enfermedad han cambiado, producto de la existencia de tratamientos e intervenciones eficaces, así como por resultado de las posibilidades concretas de que las personas pudieran curarse, prevenir enfermedades y reducir los índices de mortalidad infantil a través de hospitales y dispensarios.

Al respecto, un eje que atraviesa el libro es la imposibilidad de implementar políticas sanitarias sin un incremento adecuado en las partidas presupuestarias estatales. La autora demuestra cómo la expansión a partir de los años 1940 fue acompañada de una mayor centralidad de las áreas sociales en los programas políticos, uno de los aspectos claves que ha legado el ideario sanitario. No solo fue necesario incrementar partidas para la designación de personal sanitario e infraestructura, sino que el andamiaje burocrático se debió ampliar para sostener dicha estructura. María José Ortiz Bergia (2022) analiza la decisiva voluntad política en la expansión de la estructura asistencial y preventiva y, en forma paralela, cómo creció la burocracia. Sin injerencia estatal habría sido imposible satisfacer demandas sanitarias cada vez más complejas y en aumento. Pero, además, los cambios en la administración estatal, ya sea por ampliación de la estructura o por achicamiento, no fueron meros artilugios discursivos. El financiamiento conllevó un posicionamiento ideológico en torno a cómo pensar lo social y, en este caso, lo sanitario.

Otro tópico central en el libro es la conformación de la burocracia sanitaria. La autora en el capítulo 3 se pregunta ¿más burocracia es mejor burocracia? Y para responder al interrogante analiza los criterios de reclutamiento y permanencia del personal sanitario. Al respecto, sostiene que, para el caso cordobés, el cariz técnico de las dependencias sanitarias implicó ciertas pericias para ocupar los cargos; sin embargo, también la confianza y la lealtad hacia el gobierno de turno fueron prioritarias a la hora designar los cargos de mayor importancia. De tal modo, los criterios de elección del personal obedecieron a variables políticas antes que técnicas, y los inestables tiempos de la política limitaron las capacidades de una burocracia formada a través de su permanencia en el puesto.

Según la autora, las relaciones interjurisdiccionales también fueron importantes para explicar la expansión de la estructura sanitaria provincial, que pasó de contar con veinte establecimientos en 1930 a 120 a mediados de 1950. Es por ello que en el capítulo 7 releva los ámbitos de coordinación y complementación existentes entre las autoridades nacionales y provinciales. Así, bucea entre las conferencias de ministros de Hacienda que se organizaban todos los años, las conferencias de gobernadores y ministros y las reuniones de “los lunes” en las que los gobernadores viajaban a Buenos Aires para reunirse con el presidente y luego trasladar las medidas a sus jurisdicciones. Estas instancias de comunicación política habrían sido creaciones institucionales claves en las que durante el peronismo se distribuyó poder, se lograron consensos y se gestaron políticas sanitarias.

Los espacios de conflictos están presentes en varios apartados del libro, pero quisiera destacar uno de ellos vinculado al encarecimiento de las prestaciones sanitarias. La autora demuestra que el aumento de medicamentos, alimentos, costos laborales y enseres de los hospitales que dependían de la Sociedad de Beneficencia llevaron a sus responsables a reclamar el acrecentamiento de las subvenciones, pero la respuesta por parte de las autoridades fue la suspensión de dichas subvenciones, lo que condujo a que las organizaciones voluntariamente cedieran sus instituciones a la provincia. Si bien este procedimiento fue objeto de críticas, posibilitó ampliar las responsabilidades estatales para concretar derechos sociales. Este ejemplo, que se encuentra en el capítulo 6, permite reflexionar en la actualidad sobre cómo los gobiernos pueden apuntalar con sus acciones, no sin conflictos, áreas estratégicas para el bienestar de las personas. En tiempos signados por la polarización política, este libro habilita a reflexionar en torno a debates que no son nuevos y que remiten a terrenos de disputa política en los que la negociación es compleja e involucra a una pluralidad de actores. Asimismo, nos invita a pensar sobre el rol de los Estados, sus burocracias y las dificultades en torno a la ampliación de derechos sociales sin un involucramiento de las responsabilidades públicas.

Otro aspecto a destacar es cómo la autora incluye a las problemáticas rurales en la agenda sanitaria provincial, aspecto muchas veces olvidado en otros trabajos que se concentran en estudiar solamente la salud urbana. Según Ortiz Bergia (2022), la salubridad rural fue instituida como núcleo de acción oficial, y, como consecuencia, enfermedades como la brucelosis, el mal de Chagas y el paludismo se plasmaron en acciones concretas de las políticas provinciales tales como estímulos para radicar a profesionales en áreas alejadas de los grandes centros urbanos. La autora, si bien destaca y analiza los inconvenientes de dichas políticas, como la falta de recursos, de personal y la imposibilidad de sostenerlas en el tiempo, enfatiza también su importancia para brindar servicios de salud al conjunto de la población urbana y rural de un extenso espacio provincial atravesado por desigualdades perdurables.

Por último, quisiera destacar el uso de fuentes. La autora trabaja con datos que suelen ser difíciles de producir. Bucea entre boletines estatales, anuarios estadísticos, informes de gestión, organigramas, trayectorias de profesionales y prensa periódica nacional y provincial. Estas fuentes demandan una laboriosa y artesanal búsqueda y sistematización. Ella pudo hallarlas en diferentes reservorios físicos de la Argentina y analizarlas para contarnos esta amena historia del estado cordobés, pero que también nos permite comprender las desigualdades sociales, las diferencias en el acceso sanitario y las acciones de la sociedad civil para ampliar la concreción de derechos. Entrecruza constantemente aspectos administrativos y de gestión con trayectorias de funcionarios y en dicho enlace no se olvida ni de los problemas teóricos que implica pensar el federalismo ni de la desigualdad territorial y regional.

Celebro la publicación de *La salud como problema provincial* y estoy convencida de que abrirá puertas para seguir reflexionando sobre el diseño de las políticas públicas y los debates que están por detrás a la hora de pensar en la ampliación o el achicamiento del Estado. Tópicos que no son meramente coyunturales e implican una reflexión política y académica en torno al influjo del pasado en el presente y, por lo tanto, un nuevo posicionamiento de quienes hacemos historia para involucrarnos con el presente.
